# Possible misdiagnosis of 46,XX testicular disorders of sex development in infertile males

**DOI:** 10.7150/ijms.46058

**Published:** 2020-05-11

**Authors:** Tong Chen, Linlin Tian, Xianlong Wang, Demin Fan, Gang Ma, Rong Tang, Xujun Xuan

**Affiliations:** 1 Center for Reproductive Medicine, Shandong University, National Research Center for Assisted Reproductive Technology and Reproductive Genetics; The Key Laboratory for Reproductive Endocrinology of Ministry of Education, Jinan, Shandong 250021, P.R. China; 2 Department of Pediatric Surgery, Shanghai Children's Hospital, Shanghai Jiao Tong University, Shanghai, 200062, P.R. China; 3 Department of microbiology, Faculty of Basic Medical Sciences, Guilin Medical University, Guilin, Guangxi 541004, P.R. China; 4 Department of Urology, Shandong Provincial Qianfoshan Hospital, Jinan, Shandong 250002, P.R. China; 5 Department of Andrology, The Seventh Affiliated Hospital, Sun Yat-sen University, Shenzhen, 518107, P.R. China.

**Keywords:** 46,XX disorders of sex development, azoospermia factor, misdiagnosis, mosaicism, sex-determining region Y.

## Abstract

**Objectives:** The 46,XX disorders of sex development (DSD) is a rare genetic cause of male infertility and possible misdiagnosis of this condition has never been reported. We aim to investigate clinical characteristics and laboratory results of infertile males with possibly misdiagnosed 46,XX DSD.

**Methods:** Between January 2008 and December 2017, a retrospective case series study was performed involving sixteen 46,XX DSD males without azoospermia factor (AZF) deletion. Demographics, clinical features, laboratory results and assisted reproductive technology (ART) outcomes of these patients were depicted, and the underlying accurate diagnosis was also discussed.

**Results:** The mean age was 30.06 ± 5.40 years old. Thirteen patients (81.25%) merely obtained secondary school education. Gynaecomastia occurred in one case, and cryptorchidism appeared in two cases. Testicular volumes were equal to 15 mL on two sides in one patient who had severe asthenozoospermia. Thirteen patients (81.25%) had bilateral atrophic testes which were below 5 mL. The majority of patients were observed with elevated levels of gonadotropic hormones and decreased testosterone values. Neither AZF region nor sex-determining region Y gene was absent among all patients. Twelve patients had normal ejaculatory function, whereas four were diagnosed with ejaculatory dysfunction. Eleven patients (68.75%) were diagnosed with azoospermia. Testicular sperm aspiration was performed in six subjects (37.50%). The pathological results showed that Leydig cell hyperplasia with spermatic failure was found in each case, and no sperm was found in testicular tissue. ART with donor sperm was conducted in 15 patients. Live birth was achieved in three cases through artificial insemination by donor and in one case using in-vitro fertilization by donor.

**Conclusions:** Chromosomal analysis rarely yields 46,XX karyotype combined with no deletion of AZF in infertile males. Under this condition, molecular analysis should be conducted to avoid potential misdiagnosis and false interpretation of other findings.

## Introduction

It is estimated that approximately 10% of the male population is affected by infertility worldwide 1, and genetic disorders account for around 15% of male infertility 2. Genetic mosaicism, one type of genetic disorders, refers to the condition in which regions of tissue within a single individual have different chromosome constitutions. There are three main types of mosaicism, namely, somatic, germline, and mixed gonadal and somatic. After the Klinfelter syndrome, Y chromosomal microdeletion (YCMD) is the most frequent genetic cause of spermatogenetic impairment 3. From proximal to distal, azoospermia factor (AZF) region can be further divided into AZFa, AZFb, AZFc, and AZFd regions. Complete deletions of AZFa or AZFb region invariably result in azoospermia, whereas AZFc deletions are associated with various phenotype ranging from azoospermia to mild oligozoospermia 2.

First depicted by de la Chapelle et al. in 1964 4, 46,XX disorders of sex development (DSD) is a rare genetic cause (1:20000) of male infertility 5. Based on the 'Chicago Consensus' 6, patients with female karyotype but male phenotype are identified as 46,XX DSD. According to clinical phenotypes, 46, XX DSD can be categorized as three forms of which testicular DSD is the classical one. In addition to the discrepancy between karyotype and phenotype, positive status of sex-determining region Y gene (*SRY*) appears in about 80% of 46,XX testicular DSD males 7, 8. *SRY* refers to the gene on the Y chromosome that triggers male development. Specifically, *SRY* encodes the testis-determining factor that causes the undifferentiated gonadal tissue of the embryo to form testes 9. In 46,XX testicular DSD males, X;Y translocations may occur during paternal meiosis 8. The phenotypes of 46,XX testicular DSD males mainly depend on the presence of *SRY* and other genes, including *SOX9*, *SOX3*, *DAX1*, *WT1*, *FGF9*, and *SF1*, which are implicated in the sex determination cascade 2. Turning to the issue of clinical presentations, the majority of adult males with 46,XX testicular DSD present with short stature, normal pubic hair, gynecomastia, small testes, and azoospermia 10. Also, cryptorchidism and/or hypospadias are not rarely observed 11. To date, the fertility options for 46,XX testicular DSD males are not beyond assisted reproductive technology (ART) using donor sperm 12.

According to our survey, no previous studies have reported 46,XX testicular DSD males without AZF deletion or the possible misdiagnosis of this condition. Here, a retrospective case series study was performed involving sixteen 46,XX testicular DSD males without AZF deletion. Demographics, clinical features, laboratory results and ART outcomes of these patients were depicted and the possible underlying accurate diagnosis was also discussed.

## Materials and methods

### Study population

Between January 2008 and December 2017, a total of 183,342 men consulted the andrology department of our hospital. Among these men, 85,352 received a karyotype analysis. Male phenotype combined with 46, XX karyotype were detected in 160 patients. Clinical and genetic analysis of 144 patients with AZF deletion has been published elsewhere 12. Furthermore, the other sixteen males without AZF deletion were included in the present study. Medical records, including demographics, physical examination, laboratory tests and ART outcomes, were collected and analyzed. Laboratory results originating from outside institutions were not included in this study. The research ethical committee of the hospital identified and approved the study design. Considering that this was a retrospective observational study and the data were anonymous, we waived the necessity of informed consent obtained from the patients.

### Hormone profile

Values of follicle-stimulating hormone (FSH), luteinizing hormone (LH), prolactin and total testosterone were measured using electrochemiluminescence methods. Hormones investigated comprised FSH (normal level [nl]: 1.5-12.4 IU L^-1^), LH (nl: 1.7-8.6 IU L^-1^), prolactin (nl: 4.04-15.2 ng mL^-1^) and total testosterone (nl: 249-836 ng dL^-1^). Between 7 a.m. to 10 a.m., the collection of blood samples was done for hormone profile in subjects with overnight fast and in sitting posture for 30 minutes prior to sampling.

### Semen analysis

According to the fifth edition of World Health Organization (WHO) guidelines adopted in 2010 13, the semen analysis was conducted after 2-7 days of sexual abstinence. The semen samples were collected by means of masturbation.

### Chromosomal analysis

The chromosomal analysis was performed in adult men when at least one item of the following criteria was met: (1) severe oligozoospermia (< 5 million mL^-1^); (2) non-obstructive azoospermia (NOA) 14; (3) preparing for ART prior to therapeutic procedures; (4) having family history of malformations, intellectual disability, or recurrent spontaneous abortion 15. Routine chromosomal analysis was conducted through the G-banding method from subjects' peripheral blood lymphocytes, and at least 20 metaphases per subject were completed for routine chromosomal analysis. Chromosomal abnormalities were assessed based on the criteria proposed by the International System for Human Cytogenetic Nomenclature 16. In our hospital, when chromosomal analysis yielded abnormal results, the subject would be arranged to reproductive genetics department for genetic counselling.

### Detection of Y chromosome microdeletion and* SRY*

The detection was conducted in men with severe oligozoospermia or NOA. Eleven sequence-tagged sites (STS) from the AZF region were selected to explore the submicroscopic deletion. The STS markers were *ZFY* and *SRY* for internal control region. Samples from normal fertile male were used as positive control. Genomic DNA was extracted from peripheral blood lymphocytes according to practice guidelines. Multiplex polymerase chain reaction analysis was performed to investigate the deletion on *SRY* or AZF region by utilizing the testing system of YCMD. If STS deletions were detected, another independent test of the sample would be conducted repeatedly as described above.

### Testicular biopsy and ART outcomes

Among 46,XX DSD males without complete deletion of AZFa or AZFb region, testicular biopsy is an optional choice based on patient preference. ART outcomes were evaluated through a live birth, suggesting that viable neonate was delivered after at least 28 weeks of gestation.

### Statistical Analysis

For continuous variables, results were expressed as mean with standard deviation (SD). In addition, we described categorical variables through N and percentage. All analyses were conducted using SPSS 22.0 (Chicago, USA).

## Results

### Demographics and clinical features

The mean age was 30.06 ± 5.40 years old. In six patients (Patients 1, 2, 4, 9, 15, 16), the highest level of education was a junior high school diploma, in two (Patients 12, 14) it was a senior high school diploma and in 5 (Patients 6, 8, 10, 11, 13) it was technical secondary school diploma. In three patients (Patients 3, 5, 7) the highest education achievement was college degree. In other words, three (18.75%) achieved college degree, which was the highest level of education among these patients. Gynaecomastia occurred in one patient (Patient 12). As for congenital anomaly, bilateral cryptorchidism was detected in two cases (Patients 12, 14) who had not undergone orchiopexy. Among 14 patients without cryptorchidism, testicular volumes of one patient (Patient 15) were 15 mL on two sides, whereas 13 cases presented with bilateral atrophic testes. Among these 13 patients, left and right mean testicular volumes were 1.77 ± 0.73 mL and 1.92 ± 0.76 mL, respectively. Besides, there were no patients with history or presentations of hypospadias. Twelve patients had normal ejaculatory function, whereas four were diagnosed with ejaculatory dysfunction. More detailed information of demographics and clinical features among the patients is listed in Table 1.

### Laboratory results and ART outcomes

Genetics evaluation, including karyotype, *SRY* and AZF deletions, was conducted in all patients. Neither AZF region nor *SRY* was absent among these 16 patients. Eleven patients (68.75%) were diagnosed with azoospermia. Besides, severe asthenozoospermia was diagnosed in one (Patient 15). Testicular sperm aspiration (TESA) was performed in six subjects (37.50%). The pathological results showed that Leydig cell hyperplasia with spermatic failure was found in each case, and no sperm was found in testicular tissue. FSH values were above the upper limit among 13 cases (81.25%). Among 15 cases with available LH values, 13 patients (86.67%) had LH values above the upper limit. Three patients had no available total testosterone values, and eight patients (61.54%) had total testosterone levels below the lower limit.

ART using donor sperm was conducted in 15 patients diagnosed with azoospermia or ejaculatory dysfunction. Live birth was achieved in three cases (Patients 3, 7, 13) using artificial insemination by donor (AID). In-vitro fertilization (IVF) with donor sperm resulted in a live birth in one case (Patient 16). Specifically, the patient (Patient 15) with severe asthenozoospermia was 30 years old, and his wife was 28 years old. The couple had four years of infertile history. The medical history of the patient was unremarkable, and no malformation or hereditary disease was reported in the families. Furthermore, testicular volumes of both testes were within the normal range and so were levels of hormones (Table 2). Ultrasonography of male reproductive system was performed and both testes were normal. Semen analysis was conducted twice, and both of the two results met the criteria of severe asthenozoospermia. Neither AZF region nor *SRY* of the patient was absent. During genetic counselling, clinicians offered two options for the couple, namely, preimplantation genetic diagnosis for aneuploidy screening (PGD-AS) and ART using donor sperm. The couple opted for PGD-AS, and then one cycle of IVF treatment with intracytoplasmic sperm injection (ICSI) was performed. On the day of egg retrieval, 15 oocytes were retrieved and 14 were MII oocytes. There were two 1PN and eleven 2PN. On the third day, 9 good embryos formed but embryo transfer was not conducted. Two embryos were then frozen. We are waiting for the final pregnancy outcome of this couple, and the case report is under preparation.

## Discussion

In this case series study involving 46,XX DSD males without AZF deletion, the prevalence of this medical condition was 1.87 in 10000 infertile males. As 54.45% of the subjects during this period did not receive chromosomal analysis due to lack of the indication for genetic assessment, the prevalence of 46,XX testicular DSD males without AZF deletion might be even lower in infertile males. A nationwide study demonstrated that the prevalence of 46,XX DSD reached up to 3.5-4.7 in 100 000 males 17. However, at least to date, no studies have reported 46,XX DSD male with no deletion of AZF region.

Among 16 cases without AZF deletion, *SRY* was also present in each case. In our previous study 12, we presented a series of 46,XX disorders males with AZF deletion, and* SRY* was absent in 17.44% of these patients. *SRY* is located in the euchromatin of Yp, whereas AZF region is situated in the euchromatin of Yq. The two euchromatins connect with each other via centromere. Therefore, it remains challenging to find a possible mechanism that could explain the translocation of Yp, Y centromere, and part of Yq altogether to the X chromosome. It may be possible to have either of AZF region or *SRY* gene translocated, but unlikely both. To the best of our knowledge, multiple reasons resulted in both positive status of *SRY* and no deletion of AZF region in these patients investigated. The analytical and clinical utility of methods used for *SRY* and AZF analysis should be evaluated. Maybe the regions selected, especially for AZF analysis, shared some similarities with X chromosome and these were false positive results. Although very unlikely, this condition was still possible. The more likely scenario was that these 16 patients were not 46,XX males. In fact, they might be mosaic 46,XX/46,XY or 46,XX/47,XXY males. The rate of mosaicism in many patients is higher than expected. At least in cases of 45,X the frequency of having some mosaicism for Y chromosome material reaches 10-12% of cases and 3% appear with 45,X/46,XY 18, 19. It is very likely that 46,XX males with both AZF regions and *SRY* present had the minority of mosaicism for Y chromosome material in some tissues. The mosaicism cannot be identified by traditional chromosomal analysis but could be identified using molecular analysis, such as fluorescence in situ hybridization (FISH) analysis. Akinsal et al. 20 reported that one patient was initially diagnosed with 46,XX DSD without AZF deletion. The true chromosomal status of this patient was finally shown to be 46,XX,ish der (X)t(X;Y)(p22.3;p11.3)(*SRY*+) using FISH. Thus, without suitable molecular analysis, the false-negative results of the region on Y chromosome critical for sexual differentiation or spermatogenesis could give rise to false interpretations of the phenotype and laboratory findings. In our opinion, traditional chromosomal analysis should still be firstly considered in infertile males with an indication of genetic assessment. When neither *SRY* nor AZF region was absent in males with 46,XX karyotype, molecular analysis should be performed to clarify the accurate karyotype.

Our data indicated that testicular volumes were lower than 5 mL in 15 cases (93.75%), which was similar to those reported in 46,XX DSD males 12, 20 or 46,XX/46,XY males 21-25. In addition to testicular atrophy, the majority of patients involved in this study also presented with elevated levels of gonadotropic hormones and decreased testosterone values. Therefore, most of these patients were diagnosed with hypergonadotropic hypogonadism, which was not contradictory to the presentations in 46,XX DSD males or 46,XX/46,XY males. It has been reported that mental development was affected by extra X chromosome and each extra X chromosome decreased the intelligence quotient (IQ) by 15-16 points 26. Besides, poor educational achievement was correlated with being a 46,XX DSD male 17. Our previous study also substantiated that some neurocognitive impairment happened in 46,XX DSD males with AZF deletion 12. Similarly, the current study demonstrated that the highest education status was college degree, and no patients achieved bachelor or higher degree. The majority of patients merely obtained secondary school education. It might be explained by the hypothesized neurocognitive impairment proposed by previous studies 27.

In 46,XX DSD males with AZF deletion, azoospermia or ejaculatory dysfunction was frequently detected, and treatment options offered were limited to ART using donor sperm 12. In contrast, a number of case reports suggested that live birth could be achieved by means of ICSI using sperm from males with 46, XX/46, XY true hermaphrodite 21-25. There are mainly two hypotheses regarding the mechanism of 46,XX/46,XY 28. One hypothesis is that a nondisjunction occurs at meiosis 2 during oogenesis, contributing to the abnormal 24,XX oocyte, which in turn forms a 47,XXY zygote after fertilization with the 23,Y sperm cell. The other hypothesis is that a nondisjunction occurs during early embryogenesis. In our survey, ejaculate sperm could be detected in one case who had the potential to achieve live birth through ICSI using ejaculate sperm in the future. In the husband with azoospermia or other conditions, ART using donor sperm is optional for the infertile couple 29. Eleven patients with azoospermia and four patients with ejaculatory dysfunction received ART using donor sperm. Overall, 34 cycles of AID were conducted in 15 couples of whom three achieved live birth. Furthermore, two IVF cycles using donor sperm was conducted in two couples of whom one obtained live birth. Live birth rate of AID was merely 8.82%, which was relatively lower than previously reported rate 29.

Several limitations should be recognized in the present study. Molecular analysis was not performed to confirm our impression that these cases might be false-positive 46,XX testicular DSD males and X;Y translocations might have appeared. In addition, the nature of retrospective observational study prevented us from furthering the understanding of the accurate diagnosis in these cases. Finally, estradiol levels in serum samples were not measured using mass spectrometry, which is considered the gold standard method for quantifying estradiol levels in men 30. Despite these limitations, this is the first study suggesting the possible misdiagnosis of 46,XX DSD. We also describe the likely conditions that could account for the potential misdiagnosis.

In conclusion, our data collectively demonstrated that chromosomal analysis rarely yields 46,XX karyotype combined with no deletion of AZF in infertile males. Under such an infrequent condition, molecular analysis should be conducted to avoid potential misdiagnosis and false interpretation of other findings.

## Figures and Tables

**Table 1:** Demographics and clinical presentations of the participants

Case	Age of husband (years)	Age of wife (years)	Education	Height (cm)	Weight (kg)	BMI (kg/m^2^)	Penis length (cm)	Left testicular volume (mL)	Right testicular volume (mL)
**1**	26	27	Junior high school	176	70	22.60	5	1	3
**2**	25	23	Junior high school	162	49	18.67	5	1	1
**3**	28	30	College degree	182	90	27.17	6	2	2
**4**	28	29	Junior high school	178	60	18.94	6	2	2
**5**	31	33	College degree	170	59	20.42	6	2	2
**6**	31	30	Technical secondary school	178	71	22.41	8	3	3
**7**	27	24	College degree	165	50	18.37	5	2	2
**8**	28	28	Technical secondary school	160	90	35.16	7	2	2
**9**	31	31	Junior high school	171	81.5	27.87	5	1	1
**10**	24	23	Technical secondary school	170	95	32.87	3	2	2
**11**	29	31	Technical secondary school	168	68.8	24.38	8	1	1
**12**	41	39	Senior high school	160	56	21.88	5	0	0
**13**	27	23	Technical secondary school	168	77	27.28	3	3	3
**14**	36	37	Senior high school	155	66	27.47	3	0	0
**15**	29	28	Junior high school	170	62.5	21.63	7	15	15
**16**	30	32	Junior high school	166	50	18.14	5	1	1

**Table 2:** Laboratory results and ART outcomes of the participants

Case	Serum analysis						Semen parameters					ART outcomes	
	FSH (IU/L)	LH (IU/L)	T (ng/dL)	PRL (ng/mL)	ABO blood group		Sexual abstinence (d)	Semen volume (ml)	pH	Total sperm number		AID (cycles/live birth)	IVF by donor (cycles/live birth)
**1**	45.93	24.87	219.7	33.56	AB		8	2.8	7.5	0		7/0	0
**2**	27.75	16.51	371.7	7.97	B		4	3.8	7.1	0		6/0	0
**3**	25.23	18.18	147.5	20.64	B		4	1.6	7.5	0		5/1	0
**4**	35.77	27.83	N/A	12.15	O		3	2	7.6	0		3/0	0
**5**	38.61	23.13	153.1	9.18	O		4	3.8	7.5	0		2/0	0
**6**	36.13	21.13	299.1	N/A	B		7	2.3	7.5	0		1/0	0
**7**	35.38	21.26	52.23	N/A	AB		7	2.8	7.5	0		1/1	0
**8**	16.69	12.2	53.13	N/A	A		4	3.4	7.5	0		1/0	0
**9**	47.09	24.91	37.51	5.97	A		N/A	N/A	N/A	N/A		0	1/0
**10**	19.2	12.58	110.3	13.42	O		9	1.6	7.2	0		2/0	0
**11**	21.1	13.55	76.26	11.79	B		1	1.9	7.5	0		2/0	0
**12**	3.2	4.71	N/A	N/A	B		N/A	N/A	N/A	N/A		2/0	0
**13**	57.5	17.3	N/A	N/A	A		N/A	N/A	N/A	N/A		1/1	0
**14**	0.94	N/A	731.2	12.95	B		N/A	N/A	N/A	N/A		1/0	0
**15**	5.46	5.69	471.6	N/A	B		3	3.1	6.7	105.71		0	0
**16**	22.58	17.32	61.72	33.56	O		3	4	7.5	0		0	1/1

FSH follicle-stimulating hormone, LH luteinizing hormone, PRL prolactin, T total testosterone, ART assisted reproductive technology, AID artificial insemination by donor, IVF in-vitro fertilization
